# *S*-Propargyl-cysteine prevents concanavalin A-induced immunological liver injury in mice

**DOI:** 10.1080/13880209.2022.2080234

**Published:** 2022-06-14

**Authors:** Beilei Ma, Yicheng Mao, Lingling Chang, Tao Dai, Xiaoming Xin, Fenfen Ma, Zhijun Wang, Zhuqing Shen, Qibing Mei, Yizhun Zhu

**Affiliations:** a Shanghai Key Laboratory of Bioactive Small Molecules, School of Pharmacy, Fudan University, Shanghai, China; b China State Institute of Pharmaceutical Industry, Shanghai, China; c State Key Laboratory of Quality Research in Chinese Medicine, School of Pharmacy, Macau University of Science and Technology, Macau, China

**Keywords:** Hydrogen sulphide, cystathionine γ-lyase, autoimmune hepatitis, mitogen-activated protein kinase

## Abstract

**Context:**

*S*-Propargyl-cysteine (SPRC), an endogenous H_2_S modulator, exerts anti-inflammatory effects on cardiovascular and neurodegenerative disease, but it remains unknown whether SPRC can prevent autoimmune hepatitis.

**Objective:**

To evaluate the preventive effect of SPRC on concanavalin A (Con A)-induced liver injury and uncover the underlying mechanisms.

**Materials and methods:**

Mice were randomly divided into five groups: control, Con A, SPRC (5 and 10 mg/kg injected intravenously once a day for 7 days), and propargylglycine (PAG; 50 mg/kg injected intraperitoneally 0.5 h before SPRC for 7 days). All mice except the controls were intravenously injected with Con A (20 mg/kg) on day 7. Alanine aminotransferase (ALT) and aspartate aminotransferase (AST) levels were evaluated using kits. Inflammatory cytokines (TNF-α and IFN-γ) in the blood and in the liver were detected by ELISA Kit and real-time PCR, respectively. The expression of mitogen-activated protein kinase (MAPK) pathway proteins (p-JNK and p-Akt) and apoptosis proteins (Bax and Bcl-2) was detected using western blotting.

**Results:**

SPRC reduced the levels of AST (*p* < 0.05) and ALT (*p* < 0.01) and decreased the release of the inflammatory cytokines. Mechanistically, SPRC increased H_2_S level (*p* < 0.05) and promoted cystathionine γ-lyase (CSE) expression (*p* < 0.05). SPRC inhibited the MAPK pathway activation and the apoptosis pathway. All the effects of SPRC were blocked by the CSE inhibitor PAG.

**Conclusions:**

SPRC prevents Con A-induced liver injury in mice by promoting CSE expression and producing endogenous H_2_S. The mechanisms include reducing the release of inflammatory cytokines, attenuating MAPK pathway activation, and alleviating apoptosis.

## Introduction

The liver is an important organ and plays a key role in glucose, lipid, xenobiotic metabolism, and antioxidant defense (Mani et al. 2014). Various types of liver diseases, including inflammatory liver disease (hepatitis), plague human health. Hepatitis, which is mostly caused by a viral infection, alcohol addiction, side effects of certain drugs, or autoimmune disorders, possesses the major pathological feature of tissue infiltration with a large number of inflammatory factors. This infiltration leads to morphological deformation and function deficiency in the liver (Zenewicz et al. 2007). Hepatitis seriously threatens the patients′ health because the normal functions of the organ, such as producing bile for digestion, producing essential hormones, eliminating the toxins from the body, and controlling fat and cholesterol levels, are undermined. There are many kinds of hepatitis, and autoimmune hepatitis (AIH) is an immune-mediated liver disease that has witnessed few major advances in treatment options over the last several decades. It barely has options for patients who are either refractory to or intolerant to standard therapy, which consists of prednisone and azathioprine. Thus, it is imperative to develop novel drugs and alternative strategies for AIH prevention and treatment.

Hydrogen sulphide (H_2_S), an endogenous signalling gas, has shown significant protective effects on cardiovascular diseases, inflammatory diseases, and cellular oxidative stress response (Ekundi-Valentim et al. 2013). Physiological H_2_S is mainly produced by three enzymes: cystathionine β-synthase (CBS), cystathionine γ-lyase (CSE), and 3-mercaptopyruvate sulphur transferase (3-MST) (Hu et al. 2011). A fourth enzymatic pathway that produces H_2_S from D-cysteine by D-amino acid oxidase (DAO) has been reported (Shibuya et al. 2013). Although CSE, CBS, and 3-MST are widely expressed in various tissues and organs, CSE is the predominant H_2_S-producing enzyme in the liver (Mani et al. 2014). Additionally, several studies have proved that H_2_S, together with CSE, plays an important role in liver diseases (Fiorucci et al. 2006; Yan et al. 2013).

The concanavalin A (Con A)-induced inflammatory liver injury model is the most widely used among the currently available immunological liver injury models. Con A is a T-cell mitogen that was originally extracted from Jack bean as a plant lectin (Tiegs et al. 1992). The high affinity of the liver sinusoidal endothelial cells to Con A results in the local proliferation of T lymphocytes in the sinusoid as well as the release of a great number of cellular inflammatory cytokines, such as tumour necrosis factor (TNF)-α and interferon (INF)-γ. The released inflammatory cytokines further activate the Kupffer cells, thus causing immunological liver injury (Knolle et al. 1996). Many novel compounds and drugs that act via different mechanisms have been tested and proven to exhibit protective or preventive effects on Con A-induced inflammatory liver injury. These mechanisms include the inhibition of inflammation-related signalling pathways, protection against apoptosis, promotion of the autophagy of liver cells, and suppression of the migration of T lymphocytes and macrophages (Wang et al. 2014; Hussein et al. 2015; Liu et al. 2015; Xue et al. 2015).

Previous studies have indicated that H_2_S exerts preventive effects on inflammatory liver diseases, including Con A-induced liver injury in mice, which mainly used sodium hydrosulphide (NaHS) as the H_2_S donor (Li et al. 2005; Zhang et al. 2010; Cheng et al. 2014). NaHS, an exogenous source of H_2_S, is not ideal for potential treatment owing to its chemical instability. Once solubilized in water, NaHS releases a large amount of H_2_S spontaneously over a short period. The shortage of NaHS has severely limited the application of H_2_S for treating inflammatory liver injury in clinical practice.

*S*-Propargyl-cysteine (SPRC) is an endogenous H_2_S modulator with good fluidity and scale-up production probability. Compared with NaHS, SPRC is more advantageous and promising in terms of medicinal properties because it has a more stable chemical property. Our earlier studies have revealed that SPRC exerts immense preventive effects on cardiovascular diseases (Kan et al. 2014; Liang et al. 2014), neurodegenerative diseases (Gong, Pan, et al. 2011; Gong, Wang, et al. 2011), and gastric cancer (Ma et al. 2011). The anti-inflammatory effect of SPRC has been established in cardiac disorders (Pan et al. 2011), vascular endothelium-related diseases (Pan et al. 2012), and neurodegenerative diseases (Gong, Wang, et al. 2011). In some previously reported models, SPRC has been shown to promote the expression of CSE and the production of H_2_S and subsequently regulate cellular signalling pathways, such as apoptotic and inflammatory pathways. Furthermore, SPRC administration has been shown to inhibit the release of inflammatory cytokines (Wen and Zhu 2015). However, its effect on autoimmune hepatitis (AIH) remains unknown. Hence, this study was aimed at examining the preventive effect of SPRC on Con A-induced immunological liver injury and clarifying the underlying mechanisms.

## Materials and methods

### Reagents

SPRC was synthesized as previously described (Wang et al. 2009). Con A and propargylglycine were purchased from Sigma-Aldrich (St. Louis, MO, USA). Alanine aminotransferase (ALT) and aspartate aminotransferase (AST) activity detection kits were purchased from Nanjing Jiancheng Bioengineering Institute (Nanjing, China). Trizol reagent was purchased from Thermo Fisher Scientific (Waltham, MA, USA). RNA polymerase chain reaction (PCR) kit was purchased from Takara Biotechnology (Dalian, China). The antibodies used for western blotting of CSE, p-JNK, p-Akt, Bcl-2, and Bax were obtained from Santa Cruz Biotechnology (TX, USA).

### Animals and Con A-induced liver injury model

Male BALB/c mice (weight: 22 ± 2 g, age: 6–8 weeks) of above Grade II were obtained from Shanghai Laboratory Animal Commission (Shanghai, China). All the experimental mice were raised under specific pathogen-free (SPF) conditions (i.e., 12 h light/dark cycle; temperature 25 °C; humidity 55–60%). All mice were maintained according to the Guide for the Care and Use of Laboratory Animals of the Ministry of National Health and Family Planning of the People’s Republic of China, and all experimental protocols were approved by the Department of Animal Care and Use Committee of Fudan University. Con A was dissolved in pyrogen-free saline at the concentration of 2 mg/mL. The mice were randomly assigned to the following groups: (1) The mice in the control group were intravenously injected via the tail vein with 10 mL/kg saline. (2) The mice in the model group were intravenously injected via the tail vein with 20 mg/kg Con A. (3) The mice in the SPRC treatment groups were continuously intravenously injected with SPRC via the tail vein at 5 or 10 mg/kg for 7 days. On day 7, the mice were intravenously injected *via* the tail vein with SPRC (5 or 10 mg/kg) an hour before Con A (20 mg/kg) injection. (4) The mice in the PAG + SPRC + Con A group were first intraperitoneally injected with PAG at 50 mg/kg and, after 30 min, they were intravenously injected with SPRC at the concentration of 10 mg/kg, similar to that in the SPRC treatment group before Con A injection. The liver tissues and blood samples were collected at 8 h or 12 h after the establishment of the model.

### Histopathology

After 12 h of Con A injection, the mice were sacrificed and the liver tissues were collected for hematoxylin-eosin (HE) staining. Briefly, the liver tissues were fixed in 4% paraformaldehyde until paraffin embedding, which was performed as per the traditional method. The degree of tissue damage and inflammatory injury was recorded using paraffin sections under an optical microscope.

### Enzyme-linked immunosorbent assay (ELISA)

Blood samples from each group were collected 12 h after Con A injection, and the blood serums were collected. In the mice serum, the levels of TNF-α and IFN-γ were measured by using commercially available kits (Boatman Biotech, Shanghai; Dakewe Biotech, Shenzhen; China) according to the manufacturer’s instructions. The ultimate results were expressed as a picogram of cytokine per millilitre of the media.

### Reverse transcriptase-PCR (RT-PCR)

After 12 h of Con A injection, the mice were sacrificed and their liver tissues were collected. The liver tissues were frozen in liquid nitrogen immediately until analysis. In Con A-induced mice liver, the mRNA levels of TNF-α and IFN-γ were quantified by RT-PCR. Briefly, total RNA was extracted from the liver tissues by using Trizol reagent, as per the manufacturer’s instructions. RT-PCR was conducted in the Real-time PCR system (Bio-Rad, Hercules, CA, USA) according to the manufacturer’s guidelines. The following primers were used in this experiment:

TNF-α: (forward) 5′-ATGAGCACAGAAAGCATGATC-3′,
  (reverse) 5′-TACAGGCTTGTCACTCGAATT-3′;
INF-γ: (forward) 5′-ACAGCAAGGCGAAAAAGGATG-3′,
   (reverse) 5′-TGGTGGACCACTCGGATGA-3′;
GAPDH: (forward) 5′-GGCATCTTGGGCTACACT-3′,
   (reverse) 5′-GCCGTATTCATTGTCATACC-3′.

### Western blotting

After 12 h from Con A injection, the liver tissues were collected and frozen in liquid nitrogen immediately until analysis. The cell lysates were prepared using a RIPA lysis buffer (Beyotime Biotechnology, Shanghai, China) containing protease and phosphatase inhibitors. The proteins were analyzed by Western blotting as per the standard procedures. Briefly, the proteins were separated by electrophoresis in a sodium dodecyl sulfate-polyacrylamide gel and then transferred onto a polyvinylidene fluoride membrane. The membrane was incubated with different primary antibodies and GAPDH at 4 °C overnight after blocking. The membrane was then washed with TBST (0.1% Tween-20) and incubated with either horseradish peroxidase-conjugated goat anti-rabbit or anti-mouse antibodies for 1 h, followed by detection with chemiluminescence (ECL, Millipore, Billerica, MA, USA). GAPDH was used as the loading control.

### H_2_S measurement

The blood samples from each group were collected 12 h after Con A injection, and the blood serums were collected. H_2_S concentrations in the serums were evaluated. The mouse serums (75 μL) were mixed with 250 μL of 1% zinc acetate and 425 μL of ddH_2_O. Then, 133 μL of 20 mM *N*-xylylenediamine (7.2 M HCl) and 133 μL of 30 mM FeCl_3_ (1.2 M HCl) were added and maintained at room temperature for 10 min. After adding 250 μL of 10% trichloroacetic acid, the samples were centrifuged at 14,000 rpm for 5 min. The supernatant was removed into a 96-well plate, with three duplicates, and the absorbance was measured by a microplate reader (Infinite 1000, TECAN, Switzerland) at OD 670 nm. The calibration curve was linear from 3.125 to 250 µM NaHS.

### Statistical analysis

Data are presented as mean ± standard error of the mean (SEM). Statistical significance was calculated using a one-way analysis of variance (ANOVA). The level of significance was determined at *p* < 0.05.

## Results

### SPRC prevented Con A-induced hepatic injury

**Figure 1.**
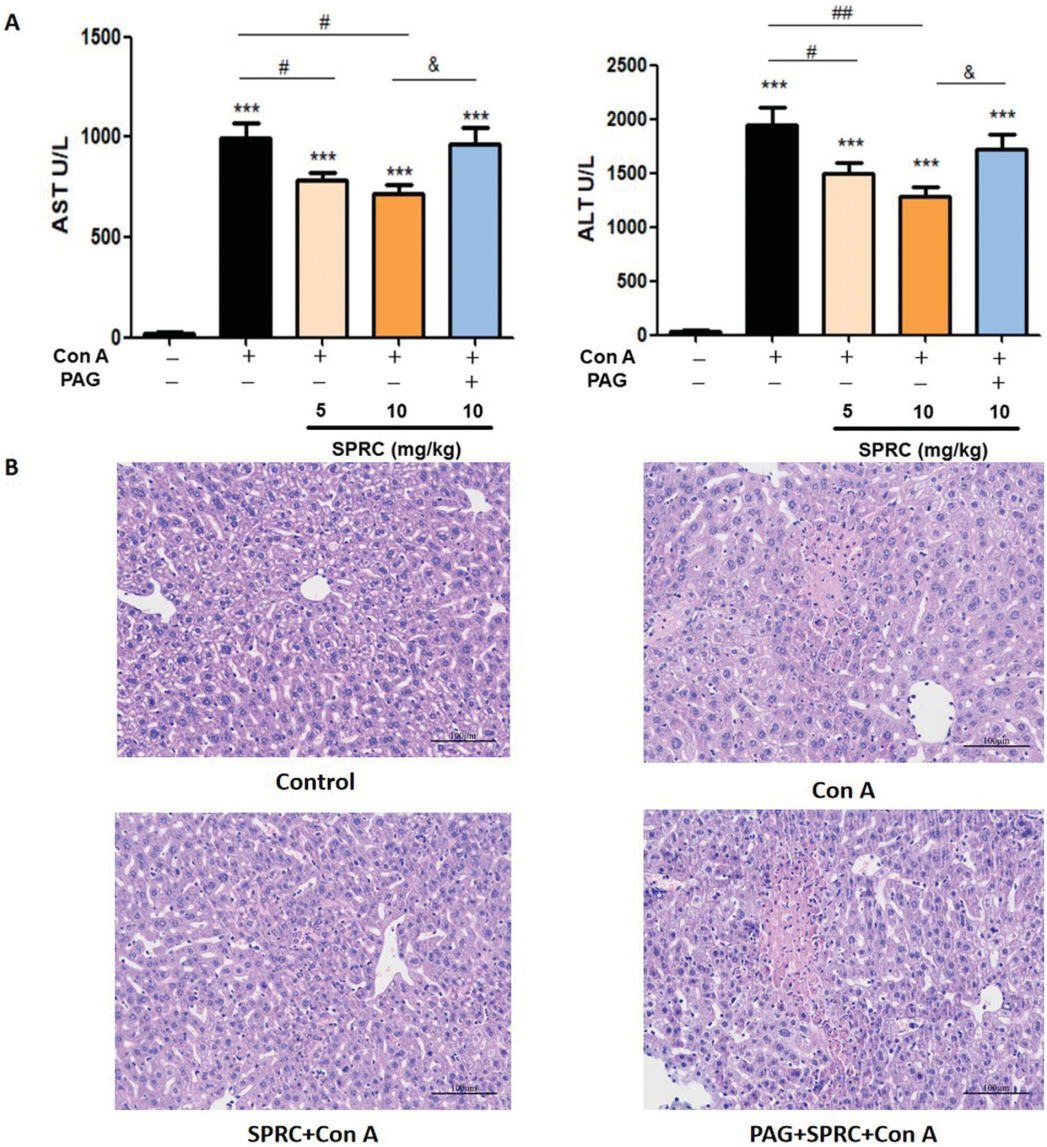
SPRC attenuated Con A-induced liver injury. (A) The AST and ALT levels of serum from Con A induction mice (*n* = 8). (B) Representative of histopathological changes in Con A-induced hepatic injury; original magnification, 200×. ***p < 0.001 *vs.* control group; ^#^*p* < 0.05; ^##^*p* < 0.01 *vs.* Con A group; ^&^*p* < 0.05 *vs.* SPRC + Con A group. Data are presented as the mean ± SEM.

Con A is often utilized in the animal models of immunological liver injury, such as acute AIH. In this study, the BALB/c mice were intravenously injected with Con A (20 mg/kg) to develop an acute liver injury. As depicted in Figure 1(A), SPRC (10 mg/kg) attenuated the serum levels of ALT (*p* < 0.001 *vs*. control; *p* < 0.01 *vs*. Con A) and AST (*p* < 0.001 *vs*. control; *p* < 0.05 *vs*. Con A) compared with those in the Con A-induced model group 8 h after the establishment of the acute liver injury model. However, the effect of SPRC was reversed by PAG (*p* < 0.05 *vs.* SPRC 10 mg/kg). H&E staining and morphological studies showed an obvious histological deterioration in the liver tissues because of Con A induction (200×) for 12 h (Figure 1(B)). The liver tissue became loose and baggy. Moreover, the hepatocytes were swollen, and the connection between them blurred. The application of SPRC significantly inhibited the histological deterioration of the liver tissue, but this preventive effect was also blocked by PAG.

### SPRC decreased Con A-induced release of inflammatory cytokines

**Figure 2.**
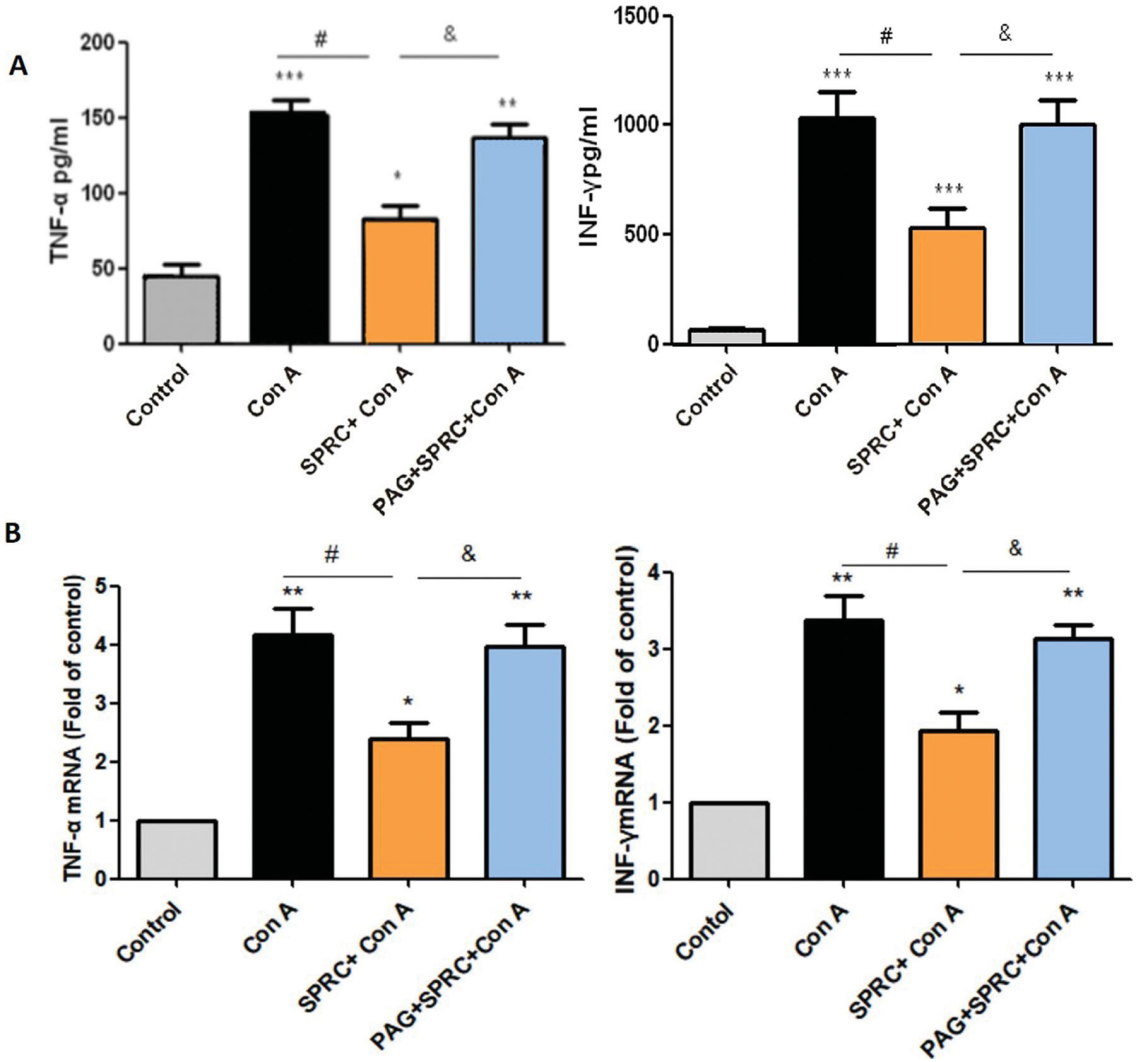
SPRC inhibited the release of inflammation factors induced by Con A in (A) the serum and in (B) the liver tissues, and the TNF-α and IFN-γ mRNA levels were normalized to the GAPDH expression in each sample (*n* = 6). *p < 0.05, **p < 0.01, ***p < 0.001 *vs.* control group; ^#^*p* < 0.05 *vs.* Con A group; ^&^*p* < 0.05 *vs.* SPRC + Con A group. Data are presented as the mean ± SEM.

Compared with the normal mice in the control group, Con A significantly elevated the levels of TNF-α (*p* < 0.001 *vs.* control) and IFN-γ (*p* < 0.001 *vs.* control) in the serum. SPRC (10 mg/kg) pre-treatment inhibited these deleterious manifestations (Figure 2(A)). Subsequently, the effect of SPRC (10 mg/kg) on the inflammatory cytokines was investigated at the mRNA level in the liver. Similar to the results observed in the serum, administration of Con A upregulated the mRNA levels of TNF-α (*p* < 0.01 *vs.* control) and IFN-γ (*p* < 0.01 *vs*. control) in the liver (Figure 2(B)). SPRC pre-treatment obviously inhibited the Con A-induced upregulation of mRNA levels. As shown in Figure 2(A,B), the CSE inhibitor PAG reversed the inhibitory effect of SPRC on the release of inflammatory cytokines in the serum and mRNA expression in the liver.

### SPRC modulated CSE and H_2_S to prevent liver injury caused by Con A induction

**Figure 3.**
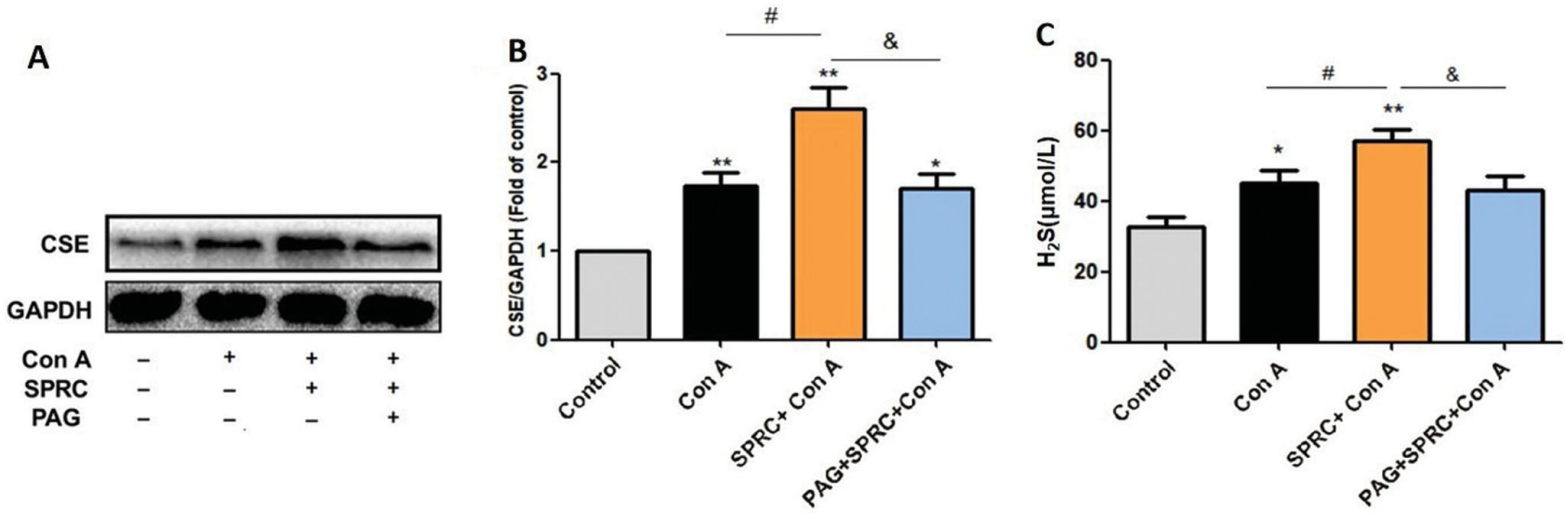
SPRC up-regulated CSE expression and consequently increased H_2_S production in Con A-induced liver injury. (A) Western blotting of the CSE protein expression. (B) The relative optical density was normalized to the GAPDH expression level (*n* = 6). (C) Serum H_2_S concentration (*n* = 6). Data are presented as the mean ± SEM. *p < 0.05, **p < 0.01 *vs.* control group; ^#^*p* < 0.05 *vs.* Con A group; ^&^*p* < 0.05 *vs.* SPRC + Con A group.

The expression of CSE in the liver was significantly increased in the Con A-induced group compared with the control group, as measured by western blotting (*p* < 0.01). With SPRC (10 mg/kg) pre-treatment, 12 h after the establishment of Con A-induced liver injury, the expression of CSE was even higher in the liver tissue than that in the Con A-induced group (*p* < 0.05). However, the high expression of CSE was constrained by PAG (*p* < 0.05) (Figure 3(A)). Accordingly, Con A resulted in a significant increase in the serum H_2_S level compared with the control group (*p* < 0.05), and SPRC pre-treatment further increased this level in the mice compared with the Con A model group (*p* < 0.05). However, the increase in H_2_S level was inhibited by the application of PAG in addition to SPRC (*p* < 0.05) (Figure 3(B)).

### SPRC inhibited the inflammatory pathway in Con A-induced liver injury

**Figure 4.**
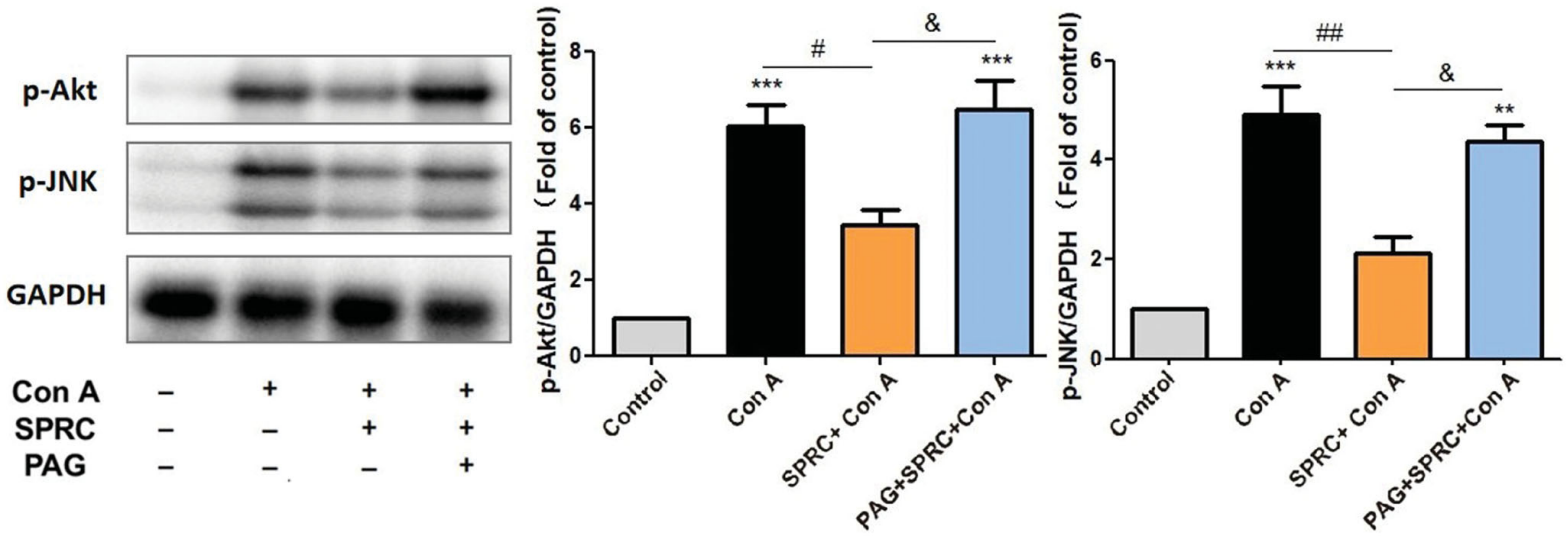
The effect of SPRC on the expression of inflammatory pathway-related proteins in Con A-induced liver injury. Western blotting of the p-JNK and p-Akt protein expression. The relative optical density was normalized to the GAPDH expression level (*n* = 6). Data are presented as the mean ± SEM. **p < 0.01, ***p < 0.001 *vs.* control group; ^#^*p* < 0.05, ^##^*p* < 0.01 *vs.* Con A group; ^&^*p* < 0.05 *vs.* SPRC + Con A group.

Previous studies have established that the release of inflammatory cytokines leads to liver injury after the Con A injection. The mitogen-activated protein kinase (MAPK) signalling pathway has previously been described to regulate the production of inflammatory cytokines. In this pathway, activation of c-Jun N-terminal kinase (JNK) and protein kinase B (Akt) has been reported to play an important role in hepatitis and determine the fate of the hepatocytes (death or survival). In this study, western blot analysis of phosphorylated JNK (p-JNK) and phosphorylated Akt (p-Akt) revealed increased expressions of these proteins in the livers of mice intoxicated with Con A after 12 h. On the contrary, SPRC (10 mg/kg) administration reduced the expressions of p-JNK (*p* < 0.01 *vs.* Con A) and p-Akt (*p* < 0.05 *vs.* Con A). This result indicated that SPRC pre-treatment suppressed the activation of the MAPK pathway (Figure 4). However, this suppression was reversed by the CSE inhibitor PAG.

### SPRC attenuated apoptosis in Con A-induced liver injury

**Figure 5.**
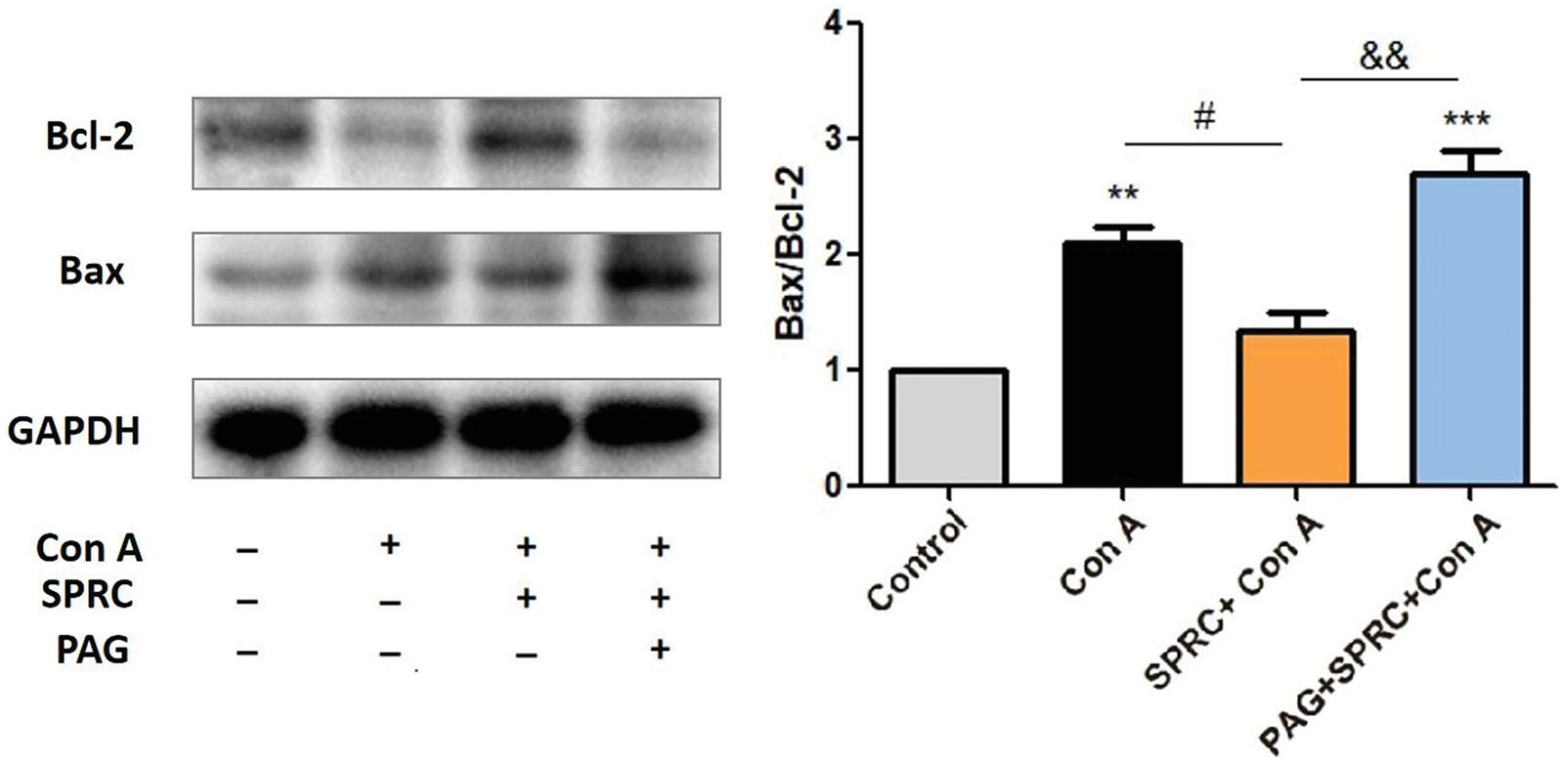
The effect of SPRC on the expression of apoptotic related proteins in Con A-induced liver injury. Western blotting of Bax and Bcl-2 protein expression. The relative optical density was normalized to the GAPDH expression level. The ratio of Bax/Bcl-2 was calculated and presented (*n* = 6). Data are presented as the mean ± SEM. **p < 0.01, ***p < 0.001 *vs.* control group; ^#^*p* < 0.05 *vs.* Con A group; ^&&^*p* < 0.01 *vs.* SPRC + Con A group.

Changes in the expression of the proteins related to apoptosis determine the viability and survival of the hepatocytes. After treating the mice with Con A for 12 h, the proapoptotic protein Bax was overexpressed, whereas the antiapoptotic protein Bcl-2 was lowered in the liver tissue. Pre-treatment with SPRC (10 mg/kg) successfully reduced the elevation of Bax in Con A-induced liver tissue and promoted the expression of Bcl-2. Again, PAG reversed the effects of SPRC on Bax as well as Bcl-2. As indicated in Figure 5, the Bax/Bcl-2 ratio was significantly elevated in the Con A-induced model group (*p* < 0.01 *vs.* control), which was significantly suppressed by SPRC (*p* < 0.05 *vs.* Con A) but promoted by PAG (*p* < 0.01 *vs.* SPRC + Con A) in addition to SPRC.

## Discussion

**Figure 6.**
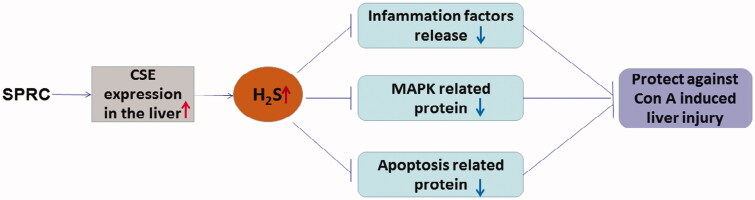
The preventive mechanisms of SPRC on Con A-induced liver injury.

Autoimmune hepatitis (AIH) seriously threatens human health; if not properly controlled, has a high tendency to develop into serious liver diseases, such as hepatic fibrosis, cirrhosis, and even hepatic cancer. The options for AIH prevention and treatment are greatly unmet, and there is an urgent need to develop new drugs and strategies. This study has investigated the effects of SPRC, a novel H_2_S donor, on Con A-induced liver injury. For the first time, this research has demonstrated that SPRC could efficiently attenuate Con A-induced liver injury to the extent of serving as a potential therapeutic candidate for AIH. This study has identified that the possible mechanisms underlying the preventive effect are the upregulation of CSE and H_2_S expression to inhibit the release of proinflammatory cytokines, reduction of the phosphorylation of crucial proteins in the MAPK signalling pathway, and inhibition of the apoptotic signalling pathway (Figure 6).

Con A-induced acute liver injury is a well-established experimental model of immune-mediated liver injury (Shao et al. 2013). Although a previous study has reported that H_2_S attenuates Con A-induced hepatitis by using NaHS as an H_2_S donor, the instability of NaHS has limited its therapeutic application. Compared with NaHS, SPRC is a more promising therapeutic candidate that can act as an endogenous modulator of CSE and H_2_S owing to its enhanced stability. SPRC has previously been considered an endogenous H_2_S-producing agent by supplying the substrate for H_2_S synthesis (Wen and Zhu 2015). This work has pioneered the research on the preventive effect of SPRC in liver injury, the results of which could extend the application of SPRC as a promising H_2_S modulator for clinical use.

Earlier studies in our lab have identified that SPRC can promote the expression as well as the activity of CSE with increased production of H_2_S, and the upregulated H_2_S has demonstrated anti-inflammatory effects in different disease models (Ma et al. 2011; Miao, Shen, et al. 2016; Miao, Xin, et al. 2016). In the present study, Con A injection increased the expression of CSE and enhanced the production of H_2_S, mainly owing to the self-protection of the body against Con A-induced inflammation injury. This upregulation of CSE is consistent with other reported inflammation models, such as cerulean-induced acute pancreatitis and lipopolysaccharide-induced endotoxemia in mice (Bhatia et al. 2005). When pre-treated with SPRC, the CSE expression and, consequently, the H_2_S production were both upregulated. Therefore, the defensive validity was further strengthened. When PAG was added, the expression of CSE and the production of H_2_S were similar to those in the Con A group, which indicated that the activity of CSE was inhibited by PAG (Figure 3). CSE is the key enzyme that produces H_2_S in the liver; moreover, H_2_S endogenously synthesized by CSE has been demonstrated to be primarily responsible for the anti-inflammatory action (Bhatia 2015). Hence, it is pivotal for SPRC to function as a modulator of CSE to control the release of H_2_S against Con A-induced liver injury.

Emerging evidence has revealed that the release of large amounts of inflammatory cytokines, including TNF-α and IFN-γ, plays an important role in the pathogenesis of the Con A-induced hepatitis model (Sass et al. 2002). Previous studies have demonstrated that antibodies against TNF-α and IFN-γ could attenuate Con A-induced liver injury (Gantner et al. 1995). Our lab-based studies have suggested that SPRC exerts an anti-inflammatory effect on cardiovascular diseases, neurodegenerative diseases, and vascular endothelium-related diseases by suppressing the release of inflammatory cytokines and modulating the NF-κB pathway (Gong, Pan, et al. 2011; Gong, Wang, et al. 2011; Pan et al. 2011, 2012). Similar to previous studies, this research has also shown that SPRC exerts an anti-inflammatory effect on Con A-induced liver injury by suppressing the expression of inflammatory cytokines, such as TNF-α and IFN-γ, in the serum and their mRNA levels in the liver.

A study has shown that TNF-α released by the immune cells stimulates TNF-receptor-1 located on the hepatocytes, thereby activating multiple signal transduction-related proteins, such as JNK (Das et al. 2009). During the process of inflammation, JNK acts as an important kinase that determines the death or survival of the hepatocytes (Nishina et al. 2004). The mice lacking either JNK1 or JNK2 have been found to be resistant to Con A-induced liver injury (Das et al. 2009). Consistent with previous studies, this study has demonstrated that Con A obviously induced the phosphorylation of p-JNK. SPRC pre-treatment significantly inhibited the phosphorylation of p-JNK (Figure 5). Altogether, this has proven that SPRC, an H_2_S modulator, holds immense potential as an anti-inflammatory agent for preventing liver injury. However, further studies are needed to reveal the underlying mechanisms of SPRC to modulate the relationship between the release of inflammatory cytokines and the activation of the MAPK pathway. This understanding is necessary because the MAPK pathway plays a critical role in the progression of inflammation in both the liver cells and the immune cells. After the induction of liver injury by Con A, mouse liver tissues are composed of liver cells and immune cells. More detailed experiments are needed to evaluate whether SPRC inhibits the MAPK pathway in the liver cells or the immune cells.

Bax and Bcl-2 represent proapoptotic and antiapoptotic proteins, respectively, and the balance between them is closely associated with the induction of apoptosis in the cells. The results from the present study have indicated that Con A increased Bax and decreased Bcl-2, thus resulting in cell death. However, the balance between the two proteins was restored by SPRC treatment, with the downregulation of Bax and the upregulation of Bcl-2 (Figure 4). The balance was once again disrupted when the mice were pre-treated with PAG before SPRC. Thus, the study findings imply that SPRC attenuates cell death and prevents Con A-induced hepatitis by preventing the intrinsic pathway of apoptosis.

In the present study, SPRC was administered to the mice before the induction of liver injury by Con A; SPRC served as a preventive drug rather than a therapeutic one, which is not always the case in the clinical treatment of diseases. Hence, it is necessary to evaluate the therapeutic effect of SPRC in the Con A-induced liver injury model, which is likely to aid in the development of SPRC as a new drug for AIH treatment in the future. Moreover, the underlying defensive effects of SPRC against inflammatory liver damage should be explored in future mechanistic studies.

H_2_S is widely involved in various physiological and pathological processes in the body. On the other hand, the CSE enzyme is widely expressed in most tissues. SPRC chiefly increases the expression and/or activity of CSE to generate H_2_S and may have multiple targets in the body. Some novel liposomal carriers to deliver SPRC to the cells and tissues have been developed in our lab. Their physicochemical, morphological, and pharmacological properties have been characterized (Tran et al. 2019). In addition to the targeted delivery of SPRC based on this liposomal formulation, it is a well-known fact that liposomal formulations are preferably delivered to the liver, which should also be beneficial in overcoming the off-target effect of SPRC on tissues other than the liver. More studies should be performed to further evaluate the preventive or therapeutic effect of the newly designed liposomal SPRC.

## Conclusions

In this paper, it is demonstrated that SPRC prevents Con A-induced liver injury in mice by promoting CSE expression and producing endogenous H_2_S. The underlying mechanisms include reducing the activation of the inflammation-related MAPK pathway, lowering the release of inflammatory cytokines, and alleviating the apoptosis of liver tissues. The results indicated the possibility of developing SPRC as a novel drug for treating AIH. However, the possible therapeutic effect and the precise mechanism of action of SPRC against Con A-induced hepatitis should be investigated further.
